# Combined resistance mechanisms leading to high-level of cefiderocol resistance among NDM-like producing *E. coli* ST167 clinical isolates

**DOI:** 10.1007/s10096-025-05166-w

**Published:** 2025-05-27

**Authors:** Mustafa Sadek, Juan Bosch Duran, Trinad Chakraborty, Laurent Poirel, NARA Network, R. Lienhard, L. Vonallmen, C. Schilt, A. Scherler, K. Lucke, M. Jutzi, M. Reichmuth, V. Slutter, P. A. Gras, B. Suter, U. Schibli, C. Fricker, S. Pranghofer, K. Graff, S. Graf, G. Greub, D. Blanc, A. Vitale, B. Lemaire, M. Fatoux, M. Tritten, T. Simonet, L. Rumebe, N. Liassine, G. Jost, M. Rosselin, N. Wohlwend, D. Schultze, K. Burren, A. Westers, M. Imperiali, L. Pozzi, D. Balzari, G. Vaninetti, C. Cirillo, V. Gaia, E. Pianezzi, G. L. Mueller, A. Jayol, C. Guyon, D. Hyden, M. Maitrejean, V. Deggi-Messmer, D. Bandeira, C. Fournier, H. Assman, C. Nusbaumer, L. Bertaiola Monnerat, J. Schrenzel, G. Renzi, A. Cherkaoui, D. Andrey, A. Nguyen, S. Emonet, M. Eyer, R. Maret, A. Belo, D. Mabillard, M. Moraz, K. Herzog, V. Gisler, E. Hitz, M. Oberle, H. Fankhauser, N. Dubey, R. Capaul, C. Guler, M. Schoenenberger, U. Karrer, F. Imeri, H. Hinrikson, F. Piran, A. Ergani, C. Andreutti, M. Dessauges, M. Aerni, T. Schmid, I. Mitrovic, E. Gruner, V. Bruderer, D. Dimitrijevic, Y. Guillod, C. Maffioli, J. Maurer, M. Michel Blanco, M. Vogel, R. Wampfler, P. Staehli, B. Schnell, C. Zehnder, V. Di Lorenzo, C. Payen, D. Boschung, L. Comte, M. Schacher, M. Brandenberger, C. Zowa, C. O. Marti, S. Trachsel, M. C. Descombes, I. Steffen, C. Kurmann, B. von Arb, M. Wehrli, B. Elmer, A. Imhof, B. Preiswerk, B. Mathis, L. Martinotti, L. Basilico, G. Togni, P. Minkova, M. Kuegler, V. Povolo, S. Droz, M. Elzi, C. Casanova, D. Goldenberger, P. Keller, C. Lang, A. Blaich, S. Schmid, B. Ivan, A. Egli, S. Mancini, O. Dubois, K. Narr, S. Schoch, S. Ellenberger, C. Castelberg, S. Seiffert, Patrice Nordmann

**Affiliations:** 1 Medical and Molecular Microbiology, Faculty of Science and Medicine, University of Fribourg, Fribourg, Switzerland; 2 Department of Food Hygiene and Control, Faculty of Veterinary Medicine, South Valley University, Qena, Egypt; 3 Department of Hygiene and Food Control, Faculty of Veterinary Medicine, King Salman International University, Ras Sudr, Egypt; 4 Institute of Medical Microbiology, Justus Liebig University Giessen, 35392 Giessen, Germany; 5 German Center for Infection Research, Partner Site Giessen-Marburg-Langen, 35392 Giessen, Germany; 6 Swiss National Reference Center for Emerging Antibiotic Resistance (NARA), University of Fribourg, Fribourg, Switzerland

**Keywords:** *Escherichia coli*, ST167, NDM, Cefiderocol, Aztreonam/avibactam

## Abstract

**Purpose:**

A series of NDM-producing *Escherichia coli* ST167 clinical isolates exhibiting resistance to cefiderocol (FDC), with no previous exposure to this antibiotic, were analyzed in this study.

**Methods:**

The antimicrobial susceptibility testing and phenotypic detection of resistance patterns (Rapid Cefiderocol NP test and MIC determination) were performed for all tested isolates. Their entire genomes were sequenced by using the Illumina MiSeq platform and high-quality reads were *de-novo* assembled using the CLC Genomic Workbench. Genome-sequence based characteristics were analyzed using bioinformatics tools.

**Results:**

All NDM-producing *E. coli* ST167 isolates showed a high level of resistance to FDC (MICs being 64 or > 64 mg/L). The chromosomally located *cirA* gene, encoding a catecholate siderophore receptor in *E. coli*, was truncated in all FDC-resistant isolates due to a frameshift mutation (S90Y), leading to CirA-deficient isolates. A four amino acid insertion (YRIN) was also identified after residue 333 in the PBP3 protein sequence of all *E. coli* isolates. Among them, a single FDC-resistant NDM-5-producing *E. coli* isolate (1006) was additionally resistant to aztreonam/avibactam (AZA MIC of 8 mg/L). When analyzed against the genome of another FDC resistant NDM-5 producing *E. coli* ST167 containing a YRIN insertion in the PBP3, and exhibiting decreased susceptibility to AZA, the broad-spectrum ß-lactamase CMY-42 was identified.

**Conclusion:**

We identified a variety of NDM-producing *E. coli* isolates exhibiting high level of resistance to FDC as a result of the combined effect of *CirA* deficiency, along with production of NDM-type enzymes. The spread of such resistance phenotype across Europe poses great concern on the clinical efficacy of this novel drug. Additionally, the identification of an FDC- and AZA-resistant NDM-5 producing *E. coli* isolate represents one of the ultimate evolutions with a possible step towards pan-resistance.

**Supplementary Information:**

The online version contains supplementary material available at 10.1007/s10096-025-05166-w.

## Introduction

Due to increasing emergence of multidrug resistance (MDR) among Gram negatives, the newly developed siderophore cephalosporin cefiderocol (FDC) is a promising antibacterial agent. Indeed, a large variety of multidrug-resistant Gram-negatives, including carbapenem-resistant Enterobacterales often remain susceptible to FDC, and promote its use in treating these infections [1, 2]. Unfortunately, reduced susceptibility or resistance to FDC have been already reported, although this novel molecule is not yet widely used in clinics [3–6]. Gaining knowledge on the mechanisms leading to acquired resistance to FDC and the circumstances that may select for it is therefore of timely relevance. It is already established that reduced susceptibility to FDC may result from combined effect of different factors such as modification of the target (PBP-3), mutations in iron transport-related protein encoding genes (*cirA* and *fiu* for *E. coli*), co-production of serine and metallo-β-lactamases such as PER and NDM ß-lactamases, as well as deletions, insertions, and amino acid substitutions in the omega loop of AmpC enzymes [7–11]. A very few therapeutic choices are available for carbapenem-resistant Enterobacterales, particulary New Delhi metallo-β-lactamase (NDM)-producing *E. coli.* Currently, cefiderocol (FDC) or aztreonam/avibactam (AZA) are frontline therapeutic agents for NDM-producing infections [12].

During the recent few years, ST167 *E. coli* has become a clinically relevant issue primarily due to its frequent linkage with carbapenem-resistant-carbapenemase producing *Escherichia coli* strains [13]. It has been classified as a"high-risk clone"because of its widespread global dissemination, high colonization potential, and its extensive MDR profile [14, 15]. ST167 has been reported from diverse geographic areas, including Asia, Europe, the Middle East, and the Americas, highlighting its global spread [15–17]. Studies have demonstrated that carbapenem-resistant *bla*_NDM_- harbouring *E. coli* ST167 strains, particularly *bla*_NDM-5_, have been isolated from various sources including human, animal, food, and environmental samples [14, 18–24]. The dissemination of ST167 is facilitated by its ability to acquire and transmit plasmids encoding resistance genes, such as *bla*_NDM_ and *bla*_CTX-M-15_, through horizontal gene transfer. Moreover, the integration of virulence factors into its genomic structure enhance its pathogenicity, survival, and ability to colonize host tissues, immune evasion, and therefore, infection severity, creating further therapeutic challenges and sustaining the global expansion of this specific ST167 clone [14, 16, 25].

In this study, we analyzed a series of NDM-like producing *E. coli* ST167 clinical isolates exhibiting a high level of resistance to FDC (> or equal 64 mg/L) with no previous exposure to FDC. Using whole-genome sequencing (WGS), we aimed to identify the potential mechanisms contributing to such high-level FDC resistance among carbapenem-resistant NDM-like producing *E. coli* clinical isolates.

## Materials and methods

### Bacterial isolates and identification

A series of FDC-resistant NDM-like-producing *E. coli* isolates (n = 7) were obtained from the Swiss National Reference Center for Emerging Antibiotic Resistance, University of Fribourg (Switzerland). Those isolates produced either NDM-5 (n = 6), or NDM-19 (n = 1). They were obtained from various clinical sources (e.g. rectal swab, deep wound, groin swab, urethral swab, and urine sample). The isolates had been recovered from patients hospitalized in Switzerland (Lausanne, Zürich, Schaffhausen, Genève, Liestal) between 2019 and 2020 (Table 1). In addition, a single FDC-resistant NDM-5-producing *E. coli* obtained from a surveillance network (Surveillance of Carbapenem-Resistant Enterobacterales [SurvCARE]) located in the state of Hesse, Germany was included in this study (Table 1). Isolates were identified as *E. coli* using EnteroPluri-test (Liofilchem SRL, Roseto degli Abruzzi, Italy).

**Table 1:** Clinical features of the FDC- resistant NDM-producing *E. coli* clinical isolates

Isolate	Date of isolation	Origin (City/Country)	Sample type	Age (years)	Sex	Infection (I)/Colonization (C)	Carba NP	NitroSpeed-Carba NP	Rapid Cefiderocol NP	Rapid Polymyxin NP	Rapid Fosfomycin NP	Immunology test (NG-5 test)/PCR
1001	Jan-19	Lausanne/CH	Rectal swab	14	M	C	+	+	+	–	–	NDM/−5
1002	Aug-19	Zürich/CH	Deep wound	67	F	I	+	+	+	–	–	NDM/−19
1003	Apr-19	Zürich/CH	Groin swab	68	F	C	+	+	+	–	–	NDM/−5
1004	May-19	Schaffhausen/CH	Rectal swab	72	M	C	+	+	+	–	–	NDM/−5
1005	Oct-19	D	Urethral swab	38	M	I	+	+	+	–	–	NDM/−5
1006	Feb-20	Genève/CH	Urine sample	41	F	I	+	+	+	–	–	NDM/−5
1007	Mar-20	Liestal/CH	Rectal swab	39	M	C	+	+	+	–	–	NDM/−5
1008	Dec-20	Zürich/CH	Rectal swab	56	F	I	+	+	+	–	–	NDM/−5

*CH* Switzerland, *D* Germany, *M* Male, *F* Female

### Phenotypic confirmation of resistance patterns and susceptibility testing

All isolates were tested using the recently developed Rapid Cefiderocol NP test as described [26], in order to determine their resistance pattern to FDC. Thereafter, FDC susceptibility testing was performed by determining MIC values with the reference BMD method using iron-depleted-CAMH broth as previously described [27]. Results were interpreted according to the latest EUCAST breakpoints (https://www.eucast.org/fileadmin/src/media/PDFs/EUCAST_files/Breakpoint_tables/v_15.0_Breakpoint_Tables.pdf) [28]. Isolates were categorized as susceptible to FDC when MICs were ≤ 2 mg/L and as resistant when MICs were > 2 mg/L. The MICs for other antibiotics including aztreonam/avibactam (AZA) were determined using reference broth microdilution in cation-adjusted Mueller-Hinton broth (Bio-Rad, Marnes-la-Coquette, France) according to the EUCAST guidelines. Avibactam, relebactam, and vaborbactam were tested at fixed concentrations of 4, 4, 8 mg/L, respectively. The reference strain *E. coli* ATCC 25922 wild-type strain was used as quality control for all testing.

Carbapenemase production was detected by using the Carba NP and NitroSpeed-Carba NP tests [29, 30], as well as the immunochromatographic NG-Test Carba5 assay (NG Biotech, Guipry, France) that detects the five major types of carbapenemases (IMP, VIM, NDM, KPC, and OXA-48) [31]. Susceptibility to colistin and to fosfomycin were evaluated by using the Rapid Polymyxin NP and Rapid Fosfomycin NP tests, respectively [32, 33].

### Whole-genome sequencing and analyses

The genomes of all isolates were sequenced using the Illumina MiSeq platform (Illumina, San Diego, CA, USA). Briefly, the total genomic DNA (gDNA) was extracted using a QIAamp DNA minikit and 98 QIAcube (Qiagen) according to the manufacturer’s instructions. a DNA library was constructed using the Nextera sample preparation with 2 × 150 bp paired end reads (Illumina, San Diego, CA, USA) according to the manufacturer’s instructions. Assembly of Illumina short reads were performed using the CLC Genomic Workbench (version 20.0.4; CLC Bio, Aarhus, Denmark). The resulting assembled sequences were analyzed using ResFinder 4.1 software (for antimicrobial resistance genes), MLST 2.0 software (for Multilocus sequence typing (MLST) analysis on the Center for Genomic Epidemiology server (http://www.genomicepidemiology.org/). The sequence raw data have been deposited at the National Center for Biotechnology Information’s Sequence Read Archive (BioProject no. PRJNA744003 and PRJNA630933).

## Results and discussion

All seven NDM-producing *E. coli* isolates used in this study showed a positive result using the Rapid Cefiderocol NP test. MIC determinations showed that all isolates exhibited a high level of resistance to FDC (MICs being at 64 or > 64 mg/L) (Table 1). Susceptibility testing for other antimicrobials revealed that all isolates showed a multidrug-resistant phenotype, with resistance to most ß-lactams and non-ß-lactams. They were also resistant to ceftazidime-avibactam, imipenem-relebactam, and meropenem-vaborbactam, as commonly observed for producers of metallo-ß-lactamase (MBL) (and particularly NDM), but remained susceptible to fosfomycin, colistin, and tigecycline (Tables S1 and S2).

Noteworthy, a single FDC-resistant NDM-5 *E. coli* isolate was found to be additionally resistant to aztreonam/avibactam (AZA), one of the ultimate therapeutic options when treating infections caused by NDM producers (Table S2). The MIC value of AZA was 8 mg/L, therefore categorizing the isolate as “resistant” when considering the ATM resistance breakpoint (> 4 mg/L according to the EUCAST guidelines). Another *E. coli* isolate (1003) showed MIC value of AZA at 4 mg/L, being further classified as ‘intermediate resistant or less susceptible’ considering the ATM susceptibility breakpoint of ≤ 1 mg/L. The reduced susceptibility or resistance to AZA in these two FDC-resistant isolates might be attributed to the synergistic effect of production of the CMY-42 AmpC-type ß-lactamase, and a PBP3 modification (target of aztreonam), as previously described [34, 35]. The identification of FDC- and AZA-resistant isolates among NDM-5 producing *E. coli* isolates is a serious clinical concern, considering that both therapies constitute last-resort options.

Carbapenemase production in all analyzed *E. coli* isolates was detected by using both the Rapid Carba NP test and the NitroSpeed-Carba NP test [29, 30]. Using the immunochromatographic NG-Test Carba5 assay (NG Biotech, France), the production of NDM carbapenemases was detected. Using PCR and sequencing of the corresponding amplicons (Microsynth, Balgach, Switzerland), the NDM-5 and NDM-19 variants were identified.

All NDM-positive *E. coli* isolates in this studty were shown to belong to Sequence Type ST167, that has been defined as a high-risk clone, currently widely disseminating worldwide [17, 34, 35]. The NDM-5-producing carbapenem-resistant ST167 *E. coli* has been identified from human, animal, and food sources in different countries including European countries as Switzerland, Germany, and Italy [14, 16, 36–40]. This international clonal strain is associated with virulence traits and diverse resistance markers, rendering it resistant to almost all available β-lactam, including carbapenems, the newly developed β-Lactam/β-lactamase inhibitor combinations (ceftazidime-avibactam and ceftolozane-tazobactam combinations, and aztreonam-avibactam). In addition to the *bla*_NDM-5_ gene, a series of other β-lactamase encoding genes were identified, including *bla*_CTX-M-15_, *bla*_CMY-42_, *bla*_CMY-142_, and *bla*_OXA-1_ among those *E. coli* isolates (Table 2).

**Table 2:** MICs and genetic characteristics of FDC-resistant NDM-producing *E. coli* ST 167 clinical isolates

Strain		Whole-genome sequencing results									MICs (mg/L)	
	β-Lactamase content	*CMY*	*CirA*	*Fiu*	*tonB*	*exbD*	*exbB*	*baeS*	*baeR*	PBP3* gene*	FDC	ATM-AVI
1001	**NDM-5**, CTX-M-15, OXA-1	–	Truncated	WT	WT	WT	WT	Q376 K, G380E, R382H	–	**YRIN**, Q227H, E349 K, I532L	> 64	0.5
1002	**NDM-19**	–	Truncated	WT	WT	WT	WT	WT	Q184H	**YRIN**, Q227H, E353 K, I532L	64	1
1003	**NDM-5**, TEM-1B	**CMY-142**	Truncated	WT	WT	WT	WT	WT	–	**YRIN**, Q227H, E349 K, I532L	> 64	**4**
1004	**NDM-5**, CTX-M-15, OXA-1, TEM-1B	–	Truncated	WT	WT	WT	WT	WT	Q184H	**YRIN**, Q227H, E353 K, I532L	> 64	2
1005	**NDM-5**, CTX-M-15, OXA-1	–	Truncated	WT	Q160P, L55 F, K105 N	I32 F, I87L	WT	WT	Q184H	**YRIN**, Q227H, I532L, E353 K	> 64	2
1006	**NDM-5**, TEM-1	**CMY-42**	Truncated	WT	WT	WT	WT	Q376 K, G380E, R382H	–	**YRIN**, Q227H, E353 K, I532L	> 64	**8**
1007	**NDM-5**, CTX-M-15, OXA-1	–	Truncated	WT	WT	WT	WT	WT	Q184H	**YRIN**, Q227H, E353 K, I532L	> 64	1
1008	**NDM-5**, CTX-M-15, OXA-1	–	Truncated	L254M	WT	WT	WT	Q376 K, G380E, R382H	–	**YRIN**, Q227H, E353 K, I532L	> 64	1

EUCAST breakpoints (2022): Cefiderocol (FDC): S ≤ 2, R > 2; Aztreonam: S ≤ 1, R > 4

The production of NDM-type MBLs contributed to of FDC resistance in our isolates. However, production of NDM alone is not sufficient to confer resistance to FDC [8]. In another study, increased copy number and increased expression of *bla*_NDM-5_ have been shown to confer FDC resistance in *E. coli* [21]. Other NDM-type enzymes have been reported to impact the activity of FDC in a similar manner [10].

The *cirA* gene that encodes a catecholate siderophore receptor in *E. coli* was found to be truncated in all those FDC-resistant isolates, as a result of a frameshift mutation (S90Y) leading to a CirA deficiency. This trait might therefore likely contribute to the high-level resistance to FDC observed for our isolates, especially when considered with the concomitant production of NDM-type enzymes [4, 41, 42].

A single amino-acid substitution, namely L254M, that had previously been shown to be involved in reduced susceptibility to FDC, was identified in the iron-catecholate transporter Fiu encoding gene of a single isolate (1008) [2]. Substitutions of *baeS* (Q376 K, G380E, and R382H) or *baeR* (Q184H) encoded proteins were also identified in three and four isolates, respectively. In addition, substitutions in the *exbD* encoded accessory protein, which is related to iron transport, was identified in one isolate (1005). In previous studies, mutations in the *baeS* gene, encoding a sensor kinase protein of the two-component BaeSR signal transduction system, were shown to be likely responsible for increased MIC values of FDC [9, 43]. In a single isolate (1005), three amino acids substitutions (Q160P, L55 F, K105 N) were identified in the TonB3 protein sequence (a component of the TonB3-ExbB3/D3 complex), which is providing energy required for FDC transport and associated with iron acquisition.

According to the current guidelines published by the European Society of Clinical Microbiology and Infectious Diseases and the Infectious Diseases Society of America, the combination of ceftazidime-avibactam with aztreonam or the use of cefiderocol are consideered as a first-line treatment for infections associated with NDM-producing *E. coli* [44, 45]. However, association of NDM-producing *E. coli* strains with resistance mechanisms to either aztreonam-avibactam or cefiderocol may complicate significantly treatment of infections associated with those resistant strains. Other alternative options such as tigecycline and eravacycline were also proposed [44, 45]. Very recently, we have shown that future therapeutic β-lactam/β-lactamase inhibitor (BL/BLI) combinations particularly cefepime-zidebactam and meropenem-nacubactam might be an excellent therapeutic alternatives against multidrug-resistant Enterobacterales exhibiting reduced susceptibility or resistance to cefiderocol [46]. Moreover, cefiderocol based combinations with several β-lactamase inhibitors such as avibactam, taniborbactam, relebectam, zidebactam, and nacubactam might be also an interesting options [46]. On the other side, rapid susceptibility testing is the key for an immediate and succesful cefiderocol-based treatment. However, it might be still challenging. Therefore, the use of rapid cefiderocol NP test [26], requiring a single methodological step, could be a useful AST tool for faster clinical decision-making and optimum antibiotic stewardship of infections. Finally, national and regional surveillance programs should be adopted to track the prevalence of FDC and AZA resistance and to establish the optimal therapeutics.

## Conclusion

Our data showed that the high-level resistance to FDC among NDM- producing *E. coli* isolates analyzed as a result of synergistic effect of CirA deficiency and expression of metallo-β-lactamases (NDM-type enzymes) may be observed not only in China [47], but also, in Europe. In addition, among those strains, resistance to AZA was observed. Association of mechanisms of resistance to novel drugs may complicate significantly treatment of infections associated to those resistant strains.

## Supplementary Information

Below is the link to the electronic supplementary material.

ESM 1 (DOCX 25.4 KB)

ESM 2 (DOCX 23.5 KB)

## Data Availability

No datasets were generated or analysed during the current study.
