# Supplementary material for: Combined resistance mechanisms leading to high-level of cefiderocol resistance among NDM-like producing E. coli ST167 clinical isolates
Source: Eur J Clin Microbiol Infect Dis. 2025 May 27;44(9):2059–66. doi: 10.1007/s10096-025-05166-w (PMC12457464; doi:10.1007/s10096-025-05166-w)
Supplement: Supplementary file 1 — (DOCX 25.4 KB) [file 10096_2025_5166_MOESM1_ESM.docx]

**Table S1: Antibiotic resistance profile of the FDC-resistant NDM-producing *E. coli* isolates.**

| **Strain** | **Ampicillin** | **Amoxicillin /Clavulanic acid** | **Ticarcillin** | **Ticarcillin/Clavulanic acid** | **Piperacillin** | **Piperacillin /Tazobactam** | **Cefotaxime** | **Ceftazidime** | **Ceftazidime /Avibactam** | **Cefepime** | **Aztreonam** | **Cefoxitin** | **Imipenem** | **Ertapenem** | **Meropenem** | **Nalidixic acid** | **Ciprofloxacin** | **Kanamycin** | **Amikacin** | **Gentamicin** | **Tobramycin** | **Trimetoprime /sulfamethoxazole** | **Tetracycline** | **Sulphonamide** | **Colistin** | **Tigecycline** | **Fosfomycin** |
| --- | --- | --- | --- | --- | --- | --- | --- | --- | --- | --- | --- | --- | --- | --- | --- | --- | --- | --- | --- | --- | --- | --- | --- | --- | --- | --- | --- |
| 1001 | **R** | **R** | **R** | **R** | **R** | **R** | **R** | **R** | **R** | **R** | **R** | **R** | **R** | **R** | **R** | **R** | **R** | **R** | **R** | S | S | **R** | **R** | **R** | S | S | S |
| 1002 | **R** | **R** | **R** | **R** | **R** | **R** | **R** | **R** | **R** | **R** | S | **R** | **R** | **R** | **R** | **R** | **R** | S | S | S | S | S | S | - | S | S | S |
| 1003 | **R** | **R** | **R** | **R** | **R** | **R** | **R** | **R** | **R** | **R** | **R** | **R** | **R** | **R** | **R** | **R** | **R** | **R** | **R** | **R** | **R** | **R** | S | **R** | S | S | S |
| 1004 | **R** | **R** | **R** | **R** | **R** | **R** | **R** | **R** | **R** | **R** | **R** | **R** | **R** | **R** | **R** | **R** | **R** | **R** | S | S | **R** | **R** | **R** | **R** | S | S | S |
| 1005 | **R** | **R** | **R** | **R** | **R** | **R** | **R** | **R** | **R** | **R** | **R** | **R** | **R** | **R** | **R** | **R** | **R** | **R** | S | S | **R** | **R** | **R** | **R** | S | S | S |
| 1006 | **R** | **R** | **R** | **R** | **R** | **R** | **R** | **R** | **R** | **R** | **R** | **R** | **R** | **R** | **R** | **R** | **R** | S | S | S | S | **R** | **R** | **R** | S | S | S |
| 1007 | **R** | **R** | **R** | **R** | **R** | **R** | **R** | **R** | **R** | **R** | **R** | **R** | **R** | **R** | **R** | **R** | **R** | **R** | **R** | S | **R** | **R** | **R** | **R** | S | S | S |
| 1008 | **R** | **R** | **R** | **R** | **R** | **R** | **R** | **R** | **R** | **R** | **R** | **R** | **I** | **R** | **R** | **R** | **R** | **R** | **R** | S | **R** | **R** | **R** | **R** | S | S | S |
