# Supplementary material for: Combined resistance mechanisms leading to high-level of cefiderocol resistance among NDM-like producing E. coli ST167 clinical isolates
Source: Eur J Clin Microbiol Infect Dis. 2025 May 27;44(9):2059–66. doi: 10.1007/s10096-025-05166-w (PMC12457464; doi:10.1007/s10096-025-05166-w)
Supplement: Supplementary file 2 — (DOCX 23.5 KB) [file 10096_2025_5166_MOESM2_ESM.docx]

**Table S2: MICs of β-lactams for FDC-resistant NDM-producing *E. coli* isolates*.***

| **Isolate** | **MICs (mg/L)** | | | | | | | | | | |
| --- | --- | --- | --- | --- | --- | --- | --- | --- | --- | --- | --- |
|  | **Cefotaxime** | **Ceftazidime** | **Ceftazidime/**  **Avibactam** | **Cefiderocol** | **Ertapenem** | **Imipenem** | **Imipenem/**  **Relebactam** | **Meropenem** | **Meropenem/**  **Vaborbactam** | **Aztreonam** | **Aztreonam/**  **Avibactam** |
| 1001 | >128 | >128 | >128 | >64 | >64 | 32 | 16 | 64 | 64 | >128 | 0.5 |
| 1002 | >128 | >128 | >128 | 64 | >64 | >32 | >32 | >32 | >32 | 1 | 1 |
| 1003 | >128 | >128 | >128 | >64 | >64 | 32 | 32 | 64 | 64 | 64 | **4** |
| 1004 | >128 | >128 | >128 | >64 | >64 | 64 | 32 | 64 | 64 | >128 | 2 |
| 1005 | >128 | >128 | >128 | >64 | >64 | 64 | 32 | 128 | 64 | >128 | 2 |
| 1006 | >128 | >128 | >128 | >64 | >64 | >32 | >32 | >32 | >64 | 32 | **8** |
| 1007 | >128 | >128 | >128 | >64 | >64 | >32 | >32 | >32 | >64 | >128 | 1 |
| 1008 | >128 | >128 | >128 | >64 | >64 | >32 | 16 | >32 | >64 | >128 | 1 |

EUCAST breakpoints (2022): Cefotaxime: S ≤ 1, R >2; Ceftazidime: S ≤ 1, R > 4; Cefiderocol: S ≤ 2, R > 2 ;Ertapenem: S ≤ 0.5, R > 0.5; Imipenem: S ≤ 2, R > 4; Meropenem: S ≤ 2, R > 8; Ceftazidime/Avibactam: S ≤ 8, R > 8; Imipenem/relebactam; S ≤ 2, R > 2; Meropenem/vaborbactam: S ≤ 8, R > 8; Aztreonam: S ≤ 1, R > 4.
